# Complementing Hi-C information for 3D chromatin reconstruction by ChromStruct

**DOI:** 10.3389/fbinf.2023.1287168

**Published:** 2024-01-22

**Authors:** Claudia Caudai, Emanuele Salerno

**Affiliations:** Institute of Information Science and Technologies, National Research Council of Italy, Pisa, Italy

**Keywords:** 3D chromatin configuration, Hi-C contact data, gene expression data, DNA binding factors, histone modification, modified bead-chain model, multiscale reconstruction algorithm

## Abstract

A multiscale method proposed elsewhere for reconstructing plausible 3D configurations of the chromatin in cell nuclei is recalled, based on the integration of contact data from Hi-C experiments and additional information coming from ChIP-seq, RNA-seq and ChIA-PET experiments. Provided that the additional data come from independent experiments, this kind of approach is supposed to leverage them to complement possibly noisy, biased or missing Hi-C records. When the different data sources are mutually concurrent, the resulting solutions are corroborated; otherwise, their validity would be weakened. Here, a problem of reliability arises, entailing an appropriate choice of the relative weights to be assigned to the different informational contributions. A series of experiments is presented that help to quantify the advantages and the limitations offered by this strategy. Whereas the advantages in accuracy are not always significant, the case of missing Hi-C data demonstrates the effectiveness of additional information in reconstructing the highly packed segments of the structure.

## 1 Introduction

By their very nature, Hi-C data (Lieberman-Aiden et al., 2009) contain information on the 3D structure of the chromatin in euchariotic cells during interphase. In each Hi-C experiment, the counts of each specific pair of genomic loci found in contact in a uniform population of cells are first debiased (Yaffe and Tanay, 2011; Imakaev et al., 2012) and then gathered in a contact frequency matrix. Even though the experiment includes millions of cells and the chromatin in their nuclei can assume different configurations, the cumulated number of contacts between all the pairs of loci is anyway indicative of the most frequent structures compatible with the data. Two kinds of approaches can then be followed when attempting to draw geometric information from Hi-C matrices. One tends to reconstruct a fiducial chromatin configuration, a sort of average structure; the other prefers to gather a population of plausible configurations, compatible with the data available. In any case, the problem is severely ill-posed, and small fluctuations in the data can lead to a large variability in the solution.

All the strategies to reconstruct the chromatin configurations need to provide a data model, that is, some relationship between the contacts and the geometry of the chromatin chain, and a solution model, that is, a mathematical entity reproducing the spatial properties of the chromatin. Some of these properties are known independently of the data, and can be used to constrain the solution once somehow included in an estimation algorithm, also accounting for the fit between the experimental and the model-generated data. As far as the data model is concerned, most popular strategies assume an explicit relationship between the number of contacts of any two loci and the Euclidean distance between them, thus transforming the chromatin configuration estimation into a classical distance-to-geometry problem. By the solution model, the chromatin chain can be represented mathematically as a purely geometric entity (piecewise linear curves, bead chain, etc.) or a physical entity, restrained by its material properties, for example, a polymer. Topological-geometric or physical properties of the solution can thus be assumed as possible constraints for the solution. Finally, the estimation algorithm translates the data model and the constraints into suitable mathematical relations to be solved for the 3D chromatin configuration. Many solutions have been proposed in the literature; for the early attempts, please refer to the bibliography in (Caudai et al., 2019a). Other recent approaches include Gong et al. (2023), who propose a nonlinear dimensionality reduction based on a divide-and-conquer approach. Vadnais et al. (2022) propose ParticleChromo3D, a particle swarm optimization approach to find the global best candidate solution. ShRec3D, proposed by Kapilevich et al. (2019), also starts by estimating a distance matrix, then combines a graph shortest path algorithm for the calculation of unknown distances and a genetic algorithm to optimise the output model. Oluwadare et al. (2018) propose 3D-max, a method where the conversion factor from contacts to distances is determined automatically through a maximum likelihood approach. Zhu et al. (2018) propose a manifold learning based framework that does not assume any specific relationship between Hi-C interaction frequencies and spatial distances, but defines a neighboring affinity represented by the probability that two genomic loci are neighbors, given by the HiC contact matrix. This method does not search for a consensus model, but uses an embedding approach to model an ensemble of chromatin conformations based on neighboring affinities and biophysical feasibility derived by a 3D polymer solution model.

Some of these solutions are still based on a contact-to-distance transformation. In our view, this is the most critical aspect concerning many reconstruction algorithms presented in the literature. Indeed, relating contact numbers to distances inevitably lead to geometric inconsistencies (Caudai et al., 2015b) and does not make biological sense either, since, whereas it is legitimate to assume that two loci with high contact frequency are close to each other, this does not mean that pairs of loci that touch sporadically are really distant. To address the inversion from frequencies to distances it is necessary to check whether the distances respect the fundamental conditions of geometric consistency, e.g., the triangular inequality. Very few papers deal with this issue. Duggal et al. Duggal et al. (2013), propose a filtering technique to select subsets of interactions obedient to metric constraints, which however has a very high computational cost. Non-violation of these conditions is a necessary but not sufficient condition for geometric coherence. If the geometric consistency conditions are severely violated, the set of distances cannot be used as a target to obtain sensible geometric conformations of chromatin. Moreover, the contacts of DNA segments inside the nucleus have both casual and functional characters and there could be physical or biochemical barriers that prevent contact; many factors and mechanisms are involved in fiber contact management by the cell, some of which have not yet been precisely identified. These are the reasons why we proposed a multiscale reconstruction method, ChromStruct, where the data model assumes directly the Hi-C contacts as cues to chromatin geometry (Caudai et al., 2015a; Caudai et al., 2019a; Caudai et al., 2019b).

Hi-C, however, is not the only experimental procedure capable of providing information about the chromatin fiber geometry. First of all, what we know about the cellular machinery is that expressed genes always correspond to DNA strands that are accessible to all the macromolecules involved in gene expression (Phillips, 2008). This means that the DNA chain in those regions must be characterized by a few contacts between loci. Conversely, unexpressed genes are normally contained in highly packed DNA strands, that is, in regions characterized by many mutual contacts. This information can be provided by RNA-seq experiments (Wang et al., 2009), which detect the DNA loci that have been transcribed. Other experiments, ChIP-seq and ChIA-PET (Li et al., 2009; Muhammad et al., 2020), analyze the interactions of proteins with DNA. In particular, these experiments locate the sites where transcription factors, other DNA binding proteins such as CTCF (Phillips and Corces, 2009) or finer molecular details such as histonic acetylation and methylation (Bannister and Kouzarides, 2011) concur to regulate the function of the genome. For our purposes, all these features are relevant in that they provide geometric information. The presence of CTCF can mark compact genomic regions, since these proteins are often (not always) associated with the formation of chromatin loops, and thus with regions characterized by high curvatures. The histonic methylation H3K27ME3, similarly, can mark compact genomic regions, since a high degree of this modification is associated with DNA regions that are rich in repressed genes. The availability of these data, thus, can immediately be useful to check whether a specific configuration, no matter how obtained, matches the expectations derived from independent data.

The same information can also be used to help a reconstruction algorithm to be more accurate. For example, Abbas et al. (2019) propose GEM-FISH, a divide-and-conquer based improvement of GEM (Zhu et al., 2018) integrating the information derived from FISH experiments. This possibility, however, should somehow be examined critically. Indeed, chromatin compactness is already represented in Hi-C information, so any additional data could just be redundant. Data redundancy can contribute to make an algorithm more robust but, in the case where gene expression, CTCF and methylation are added to Hi-C, this should be verified experimentally: a robust algorithm is not necessarily accurate. An advantage can probably be obtained in those regions, such as the chromosome centromeres, where the Hi-C data are normally missing. In our case, genomic resolution is fundamental to foresee how our additional data can help the reconstruction. Typically, depending on the restriction enzyme used in the Hi-C experiment, the maximum resolution at which the Hi-C matrices can be obtained is a few kilobase-pairs, whereas RNA-seq, ChIP-seq and ChIA-PET experiments can offer resolutions of a few base-pairs. So, the latter can be useful to complement the information at very small scales, but their effect is not so relevant at larger scales. An increase in accuracy can thus be expected in the finest details, whereas the chromatin properties observed at coarser scales, as happens with multiscale reconstruction algorithms, would only be dominated by Hi-C.

To check the validity of these considerations, we developed a new version (4.3) of ChromStruct, accepting additional inputs from CTCF binding sites, H3K27ME3 methylation sites and active genes regions^1^. A preliminary experimentation, reported in (Caudai et al., 2021b), demonstrated that histone methylation and CTCF-mediated coupling data can improve the 3D reconstruction by ChromStruct at the finest scale. To rely on those results, however, some aspects of the experimental procedure should be first validated. As mentioned, we use ChromStruct to generate a population of plausible chromatin configurations, thus mimicking a real Hi-C experiment performed on a population of cells whose nuclei do not show a single chromatin configuration, but all of them contribute to the final Hi-C matrix. We evaluate the data fit of our solutions by comparing the input Hi-C matrix with the one obtained by cumulating the contacts found in our estimated configurations. Now, there are two aspects in building this estimated Hi-C matrix that should be taken care of. First, checking whether two loci are in contact entails finding a distance threshold below which the two loci are considered in contact; second, deciding how many realizations of the chromatin configuration are statistically sufficient to say that our reconstructed matrix actually approximates the input Hi-C data. None of these necessary precautions was considered to find the results in (Caudai et al., 2021b). In this paper, we report our empirical strategy to validate those results. Furthermore, the conjecture that adding concurrent information to Hi-C would be particularly useful when some data are missing was never verified experimentally. Some of the experiments reported here deal with this problem. Section 2 briefly describes the algorithm we use, Section 3 shows the results obtained and Section 4 concludes the paper.

## 2 Methods

**FIGURE 1:**
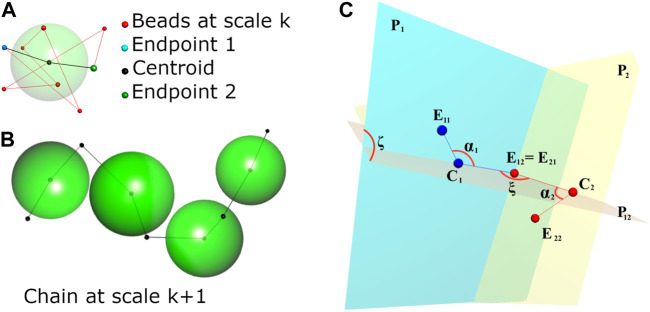
After Caudai et al. (2019b). **(A)** An illustrative example of the bead chain at some scale represented as a single, modified bead at the immediately coarser scale. The modified bead is characterized by the two endpoints of the reconstructed chain, its centroid and its physical size, represented by the semi-transparent large sphere. This allows us to adequately place and rotate each bead when reconstructing the chain at the coarser scale. **(B)** A chain at the immediately coarser scale made of four beads, properly placed and rotated. **(C)** Geometric relationships between two consecutive beads. The two beads determine, respectively, planes **P**_**1**_ and **P**_**2**_, and the two centroids **C**_**1**_ and **C**_**2**_ with the common endpoint **E**_**12**_≡**E**_**21**_ determine plane **P**_**12**_. Angles $\zeta =\hat{P_{1}P_{12}}$ and $\xi =\hat{P_{1}P_{2}}$ are used to establish the 3D rotations of the two beads. Angles *α*_1_ and *α*_2_ are fixed when modeling the beads at the immediately finer scale.

Besides being ill-posed, the problem presented above is also very large if applied directly to an entire chromosome or, even more so, to the entire genome. Finding efficient procedures to solve it is thus essential. The first observation that led us to develop our method is the fractal structure characteristic of the mammalian genomes: at all observable scales, the chromatin structure seem to be made of isolated compact regions separated by looser segments. At 100-kbp scales, these compact regions are the so-called topological association domains (TADs, Dixon et al., 2012), and similar structures are found at both smaller and larger scales. Since these structures are characterized by many internal interactions and very few contacts with the rest of the genome, their individual configurations are mainly determined by the corresponding diagonal blocks in the Hi-C contact matrix. Trieu et al. (2019) exploited this feature by proposing a hierarchical algorithm to reconstruct 3D chromosome structures starting from high-resolution data (5 kb) and using low-resolution models to fit the partial high-resolution reconstructions. This algorithm is also based on a frequency-distance conversion. Conceiving our algorithm (Caudai et al., 2015a), we also exploited the existence of these TAD-like structures. We decompose the problem by extracting these substructures from the whole smallest-scale sequence and reconstructing separately their configurations, modeled as bead chains. We thus need to solve a number of relatively simple problems rather than a much larger and complicate one. All the configurations estimated at the smallest scale are then modeled as single beads in a coarser scale chain (each bead has now the genomic size of the corresponding substructure), and used to model the entire structure at a larger scale. This new model is in turn decomposed as above, on the basis of the appropriately binned Hi-C matrix, and the single reconstructed domains are used repeatedly to build models at still larger scales until no more decomposition is possible, that is, until the binned matrix is only composed of one large block plus possibly other blocks not exceeding a fixed minimum size. Note that, from the second scale level on, the genomic size of the individual fragments is no more fixed, since each block corresponds to a specific TAD-like structure, whose size is not constant. Once the largest scale model is reconstructed, the particular model we use to represent our beads (see below) allows us to replace them with the corresponding chains at finer scales to finally obtain the whole configuration at the original genomic resolution. Despite the need for this final reconstruction, that is, another iteration throughout all the scale levels, this way of partitioning the problem is far less costly that treating all the data together. In synthesis, our solution model is a modified-bead chain, where, at each scale, each bead corresponds to an isolated domain and its structure permits its position and orientation in space to be tuned to reconstruct the whole chain at that scale. The essential geometry of the solution model is demonstrated in Figure 1. The model let the beads partially interpenetrate each other through function (Eq. 7) below, and each bead is given an approximated physical size. At the smallest scale, we only know the genomic size of each bead, and its physical size is estimated from the number of internal contacts in the corresponding matrix block: the more internal contacts, the smaller the bead. At the successive scales, each bead models a spatial configuration of beads endowed with proper sizes and mutual distances: its approximate size is estimated through the eigenvalue of the first principal component of the spatial distribution of the corresponding smaller-scale beads. The details are presented in (Caudai et al., 2019a). The approximate bead size is used to evaluate a reference ‘minimum’ distance between any two beads, denoted by $D_{i,j}^{\min}$ in the equations below, and to avoid excessive interpenetration between them. Having approximate physical sizes also allows us to enforce automatically curvature constraints along the chain and to have a final solution equipped with physical dimensions, as opposed to the methods that do not consider dimensions or derive them *a posteriori*, for example, using FISH distances or by fitting the reconstructed chain into the nucleus size.

The core of our method is thus the reconstruction of each isolated structure. As mentioned, we assume directly the Hi-C contacts as input data, by finding the pairs of beads with number of contacts larger than a threshold and favoring them to be in close proximity, and leaving all the other beads only subject to the requirement of being connected with the whole chain and non interfering with each other. The requirement of contact between pairs is enforced flexibly by a cost function of the form

$$\Phi_{\mathrm{HiC}}\left(C\right)=\underset{i,j\in L}{\sum }n_{i,j}D_{i,j}^{\min}{\left[d_{i,j}-1\right]}^{2},$$ (1)

where C represents the structure to be reconstructed, that is, the spatial coordinates and 3D rotations of all the beads, *i* and *j* are the indices of a generic pair of beads in the set L of all the pairs assumed in contact, and *n*_*i*,*j*_ and *d*_*i*,*j*_ are, respectively, the number of contacts and the physical distance between beads *i* and *j*, both normalized by $D_{i,j}^{\min}$ , obtained as the sum of the approximate radii of beads *i* and *j*. As can be noted, function (Eq. 1) penalizes quadratically the configurations where the normalized distances *d*_*i*,*j*_ are much larger than 1, and the strength of the penalization per pair is also weighted by the corresponding number of contacts: the more the contacts, the stronger the penalization assigned. Normalized distances smaller than 1 denote partially interpenetrating beads. From (Eq. 1), this condition is not strictly prohibited: interpenetration is permitted, but is only slightly penalized. $\Phi_{\mathrm{HiC}}\left(C\right)$ does not prevent two consecutive beads from interpenetrating significantly: this will be obtained by enforcing topological constraints.

As anticipated, here we try to validate the idea that the information about the strictly and loosely packed regions of DNA can help the reconstruction by complementing, Hi-C information. The CTCF data are used to modify the Hi-C data fit (Eq. 1): they are translated into a binary matrix of the same size as the input matrix, with entries equal to 1 in the detected CTCF binding sites and zero elsewhere. This matrix is then multiplied by a scalar factor and added to the Hi-C matrix to favor bead proximity in binding sites. Consequently, the combined Hi-C and CTCF data fit function becomes

$$\Phi_{\mathrm{HiC-TF}}\left(C\right)=\underset{i,j\in L^{\prime }}{\sum }\left(n_{i,j}+TF_{i,j}\right)D_{i,j}^{\min}{\left[d_{i,j}-1\right]}^{2},$$ (2)

where *TF* is the matrix described above and the symbol $L^{\prime }$ replaces L to mean that some additional bead pairs are possibly included in the set L , which would not belong to it on the basis of the Hi-C data alone. Note that by letting $n_{i,j}^{\prime }=n_{i,j}+TF_{i,j}$ Eq. 2 assumes exactly the form (Eq. 1). Considering the typical Hi-C contact frequencies found in the experiments reported by Caudai et al. (2021b), the scalar multiplier assigned to *CTCF* was fixed by trial and error to 100.

As far as the ChIP-seq and RNA-seq data are concerned, we experimented with two terms, Φ_*ChIP*_ and Φ_*RNA*_, to promote strict and loose packing, respectively:

$$\Phi_{\mathrm{ChIP}}\left(C\right)={\left[\underset{i,j\in L_{\mathrm{ChIP}}}{\max}\left(D_{i,j}^{\min}d_{i,j}\right)-D_{\min}\right]}^{2},$$ (3)

$$\Phi_{\mathrm{RNA}}\left(C\right)={\left[\underset{i,j\in L_{\mathrm{RNA}}}{\min}\left(D_{i,j}^{\min}d_{i,j}\right)-D_{\max}\right]}^{2},$$ (4)

where $L_{\mathrm{ChIP}}$ is either the set of all pairs in the chain, if the corresponding block is interested by the histone modification H3K27ME3, or the empty set otherwise; $L_{\mathrm{RNA}}$ is either the set of all pairs, if the block is included in or includes expressed genes, or the empty set otherwise; *D*_min_ and *D*_max_ are, respectively,

$$D_{\min}=D_{c}\frac{\rho^{6/5}}{6\pi },$$ (5)

$$D_{\max}=D_{c}\frac{\rho^{6/5}}{3\pi },$$ (6)

where *D*_*c*_ is the diameter of the chromatin filament (30 nm, see Hansen et al., 2018) and *ρ* is the genomic size in kbp of each chain element. Note that *ρ* has a unique meaning only at the smallest scale, since the blocks at larger scales have not constant sizes. On the other hand, as noted in the Introduction, including ChIP-seq and RNA-seq data is only significant at the smallest scale. For each configuration C , factors (Eqs. 3, 4), when present, add some positive contribution to the cost Φ_*HiC*_, thus penalizing more or less the packing of the chain. Indeed, the penalization from Φ_*ChIP*_ is minimum when the maximum distance between two beads is close to *D*_*min*_, assumed as the minimum possible size of a single bead; in turn, the minimum penalization from Φ_*RNA*_ is reached when the minimum distance between two beads is close to the maximum possible size, *D*_*max*_, of a bead.

**FIGURE 2:**
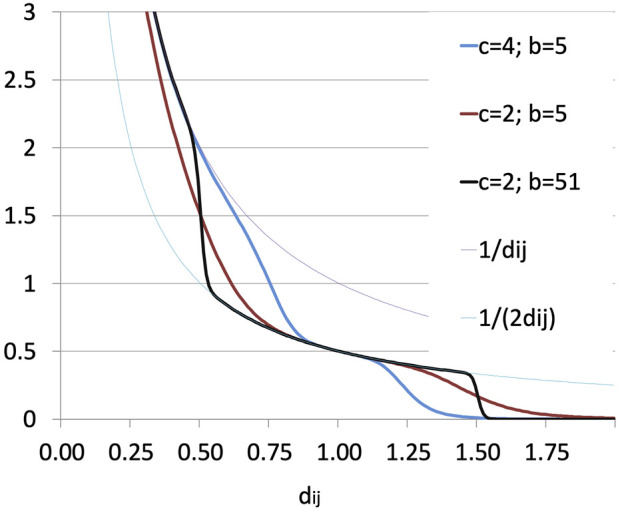
Contribution of the generic pair (*i*, *j*) to the topological constraint function Ψ(C) .

The term enforcing non-interpenetration (Caudai et al., 2019a),

$$\Psi \left(C\right)=\underset{i,j\in C}{\sum }\frac{1}{2d_{i,j}}\left[1-\frac{{\left\{cD_{i,j}^{\min}\left[d_{i,j}-1\right]\right\}}^{b}}{1+{\left\{cD_{i,j}^{\min}|d_{i,j}-1|\right\}}^{b}}\right],$$ (7)

depends on the two parameters *b*, an odd integer, and *c*, a positive constant with the dimensions of a reciprocal of a distance. For each pair (*i*, *j*) and for *d*_*i*,*j*_ ≫ 1, Ψ(C) goes rapidly to zero, that is, the relative positions of far apart bead pairs are not penalized; for *d*_*i*,*j*_ in a range around 1, the corresponding term in Ψ behaves as 1/(2*d*_*i*,*j*_), whereas for *d*_*i*,*j*_ sufficiently less than 1 the correponding term in Ψ behaves as 1/*d*_*i*,*j*_, that is, distances between elements that are significantly smaller than $D_{i,j}^{\min}$ are strongly penalized, so the two beads are only partially permitted to interpenetrate. Parameter *c* tunes the width of the interval of *d*_*i*,*j*_ around 1 where the reciprocal positions of two beads are penalized moderately; the smaller *c*, the larger that interval. Parameter *b*, in turn, tunes the slope of the two transitions that delimit the moderate-penalization interval; the larger *b*, the steepest the transitions. The influence of the generic pair (*i*, *j*) on Ψ is visualized in Figure 2 for some values of *b* and *c*.

Globally, the cost function we try to optimize to reconstruct the 3D structure of each subchain is thus the following:

$$\Xi \left(C\right)=\Phi_{\mathrm{HiC-TF}}\left(C\right)+\mu_{1}\Phi_{\mathrm{ChIP}}\left(C\right)+\mu_{2}\Phi_{\mathrm{RNA}}\left(C\right)+\lambda \Psi \left(C\right),$$ (8)

where the positive parameters *μ*_1_, *μ*_2_ and *λ* are used to tune the mutual influences of the different components of the cost function. For the time being, as made with the multiplying factor contained in *TF*, we are estimating *μ*_1_ and *μ*_2_ by trial and error. The results presented here are obtained with both fixed at 1. Searching for optimal values is deferred to the future. Unlike *μ*_1_ and *μ*_2_, which are only active at the finest scale, parameter *λ* works at all the scales, and its value cannot be kept fixed. We tune it on a predefined ratio between the weights of the data and the prior parts of the cost function. This implies a new evaluation of *λ* at each change of scale. The procedure is detailed in (Caudai et al., 2019a). The minimization strategy is based on an approximated simulated annealing where the chain evolution is obtained through quaternion operators. The Hi-C matrices derive from experiments with millions of different cells, so it is expected that many different configurations are compatible with the data. For this reason, the objective landscape is characterized by many near-optimal solutions. This is useful to our aim to find a population of different configurations and justifies our approximation of the annealing scheme. To assess the results, we evaluate the contact matrix resulting from the reconstructed configurations: for each reconstructed chain, the bead pairs that are closer than a certain threshold are considered to be in contact, and the appropriate entries of the estimated matrix are incremented by one. Cumulating the results of this procedure for all the configurations, we simulate an actual Hi-C experiment. The matrix thus obtained can then be compared to the original Hi-C matrix, for example, by using a measure of correlation.

## 3 Results

In (Caudai et al., 2021b), an early set of experiments was presented to validate the effectiveness of function Ξ(C) for reconstructing the structure of the chromatin chain and to check the actual improvement obtained by leveraging ChIP-seq, RNA-seq and CTCF data. As expected, using the additional information only proved to be advantageous at the finest scale. Indeed, the experimental results just demonstrated a slight advantage in terms of closeness between the estimated and the input contact matrices, and not for all the diagonal blocks tested. In any case, very small differences were found between the results obtained at the finest scale from the complete Hi-C data and the same data complemented by the additional information.

Before starting a new experimental phase, those results were further investigated and validated. The estimated matrices presented in (Caudai et al., 2021b) were all generated using a number of configurations fixed arbitrarily to 100. An issue of stability arises immediately: it must be decided whether 100 configurations per matrix are statistically sufficient to model the real data.

**FIGURE 3:**
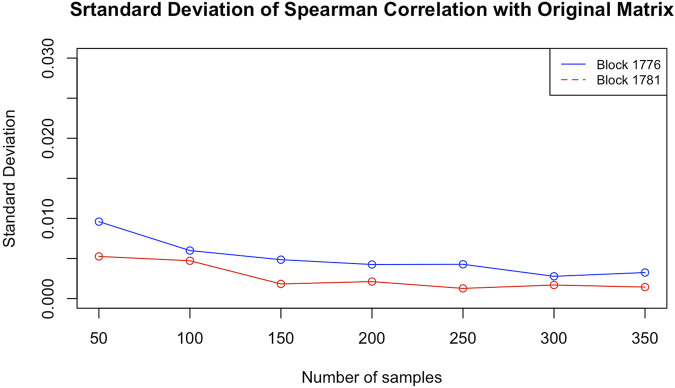
Standard deviations of the Spearman correlations between the estimated contact matrices and the experimental Hi-C matrix for blocks 1781 (loose) and 1776 (packed), as functions of the number of configurations per matrix.

To answer this question, we chose two blocks from the same data used in that paper^2^. Both are taken from chromosome 12. To check whether the particular configuration affects the result, one of them (block 1776, from 113,255 to 113,335 kbp) is highly packed, that is, with many contacts (383) in the corresponding matrix, and the other (block 1781, from 113560 to 113,645 kbp) is one of the loosest, with very few contacts (just 66). Using ChromStruct 4.3, we generated 3,500 configurations for each block. From these configurations, we generated 7 contact matrix distributions, obtained with increasing numbers of configurations (50, 100, 150, 200, 250, 300 and 350). Each distribution is drawn from 20 contact matrices, each built randomly from within a population of, respectively, 500, 1,000, 1,500, 2,000, 2,500, 3,000 and 3,500 configurations. For example, the distribution of contact matrices built by 100 configurations was obtained by 20 combinations of 100 over 1,000 different configurations. The matrices thus obtained were compared among themselves and to the original Hi-C blocks using the Spearman correlation as the similarity measure. In both the cases of presence and absence of additional data, and with no significant differences between the two blocks, the variance of the results did not decrease when using no less than 100 different configurations (see Figure 3). This means that our results obtained using 100 configurations are actually stable.

**FIGURE 4:**
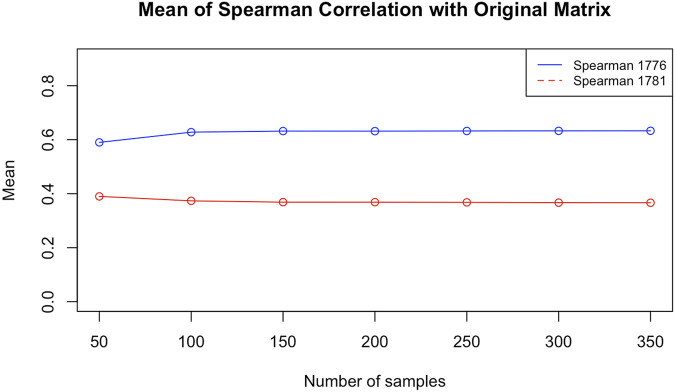
Mean values of the Spearman correlations between the estimated contact matrices and the experimental Hi-C matrix for blocks 1781 and 1776, as functions of the number of configurations per matrix.

Comparing the synthetic contact matrices to the original ones, we noticed that the results obtained for the very sparse block are less similar to the original than the ones obtained for the other block, probably because the data in the former case contain less specific information and the solution relies more on the generic prior Ψ (see Figure 4).

The results in (Caudai et al., 2021b) are thus validated for the case where the complete Hi-C data are complemented with CTCF binding sites, H3K27ME3 methylation sites and active genes locations. As observed, since the Hi-C contact data already contain all the geometric information needed to estimate the compatible configurations, possible inaccuracies apart, the presence of the additional data are often redundant, so no significant improvement can be expected. However, as anticipated in the Introduction, the situation can change if the Hi-C data are missing for significant genomic extensions. Besides validating our previous results, the aim of this paper is to check whether a significant advantage can be obtained in the case of missing Hi-C data. To this purpose, a series of simulations has been performed, based on the known CTCF binding sites. If the Hi-C data are missing in a neighborhood of two genomically distant but physically close loci, ChromStruct would only be able to enforce chain continuity and the available topological constraints there, thus potentially failing to reconstruct that neighborhood correctly. If a CTCF site is present in its vicinity, however, its knowledge can be leveraged to recover the missing information. Our simulations first consisted in selecting all the Hi-C blocks containing CTCF sites in the portion of chromosome 12 ranging from 111.5 to 115 Mbp. Then, the entire rows and columns of each block, in a neighborhood of two loci around the CTCF site, were artificially removed. Table 1 synthesizes the results of these experiments. The estimated contact matrices were obtained from 100 output configurations. It can be seen that in most cases the use of the CTCF information where there is a neighborhood of missing data allows the reliability of the reconstruction to be increased; in some cases, the result obtained using the CTCF sites almost reaches the one obtained using the whole Hi-C data.

**TABLE 1:** Spearman correlations between the original and estimated Hi-C blocks containing CTCF sites, obtained from full data, artificially truncated data and artificially truncated data plus CTCF data.

Block (kbp)	CTCF site (kbp)	CTCF site (kbp)	ECM^a^ full data	ECM^a^ missing data	ECM^a^ missing data + CTCF data
**111754–111854**	111784	111824	0.43	0.31	0.31
**111845–111915**	111875	111880	0.47	0.31	0.37
**111980–112065**	112005	112025	0.47	0.42	0.41
**112815–112865**	112850		0.43	0.15	0.21
**113440–113505**	113500		0.54	0.41	0.48
**113560–113645**	113600		0.37	0.31	0.35
**113885–113948**	113895	113905	0.36	0.18	0.34

^a^ ECM, stands for Estimated Contact Matrix (made from 100 configurations).

Using gene expression data in this case produced results that, so far, are not easy to interpret. Our first experiments on single blocks at the smallest scale show results that are not significantly different from those obtained using Hi-C and CTCF, with sometimes better sometimes worse correlations with the original matrix. At the smallest scales, however, expressed genes often extend beyond the boundaries of the isolated DNA segments, so some more convincing result could be obtained by analyzing the outputs at larger scales. A new series of experiments is scheduled to verify this conjecture. As far as methylation is concerned, it seems that its co-occurrence with the presence of CTCF binding sites is quite rare (Damaschke et al., 2020; Nanavaty et al., 2020). In fact, none of the blocks we extracted for our experiments includes both CTCF and H3K27ME3 sites, so at present we cannot assess their possible joint effect. Extending our inquiry on other parts of the human genome could give us this possibility in the future. Finally, the choice of the hyperparameter in *TF*, as well as of *μ*_1_, *μ*_2_ and *λ*, still deserves a more exhaustive assessment.

## 4 Discussion

This paper reports some experimental results obtained by the chromatin structure reconstruction code ChromStruct 4.3, open-source software with an easy-to-use graphical user interface (Caudai et al., 2021b), to check whether the use of gene expression, transcription factors and histone modification data can improve the solution with respect to the Hi-C data originally assumed as the only input. The multiscale approach adopted allows data at different genomic resolutions to be processed, and also additional information specific to selected scales to be leveraged. This is the case with the additional data considered here, which are relevant at the smallest scales typically made available by the Hi-C experiments. Possible differences with respect to the exclusive use of Hi-C data were thus expected in the fine details of the chromatin chain, and not in its global structure. Some preliminary experiments confirmed this feature, highlighting, however, that adding data is not always advantageous. The experiments reported here aim at further validating this result and exploring cases where the use of those extra-Hi-C data can actually improve the final result.

Our experimental strategy consisted in selecting cells for which Hi-C data as well as RNA-Seq, ChIP-seq and ChIA-PET data are available, then selecting parts of a single chromosome to run the experiments. Since the additional data are expected to affect significantly the finest details of the reconstructed chain, we did not run ChromStruct up to the estimation of the entire chromosome but to just reconstruct the TAD-like blocks in which the entire chain is split to implement the multiscale strategy. The results are evaluated by comparing the original Hi-C matrix blocks to the ones obtained by cumulating the contacts detected in a population of reconstructed sub-chains generated by ChromStruct.

The results obtained validated the conclusions by Caudai et al. (2021b) and confirmed that the use of additional data can even degrade the quality of the reconstructions in several cases. Indeed, when clean and complete Hi-C data are available, they contain all the needed geometrical information to estimate the chromatin structure, and adding gene expression or other structural cues only increases redundancy. This is not the case in the presence of particularly noisy or missing Hi-C data, when the additional data available can provide an important complement to the geometrical information. Our experimental results obtained after removing artificially the Hi-C data in neighborhoods of CTCF binding sites demonstrate that using the data fit function (Eq. 2), which includes CTCF data, rather than the pure Hi-C version (Eq. 1) can effectively recover the missing information. Of course, this cannot be taken as a general result even though it has been verified in several blocks from the part of chromosome under study. Genomic data are sometimes incomplete, sometimes contain bias, and when coming from different experiments could not be in perfect agreement. Sometimes, Hi-C results may disagree with RNA-seq, CHiP-seq and CTCF. Our cost function is structured to accommodate and strengthen concordant information, integrate and complete complementary information, and manage discordant information so as to reduce its influence on the result. Data coming from independent experiments are certainly an added value and an opportunity to carry out important checks on the reliability of the data available. Any estimation algorithm capable to take this wealth into account promises to provide solutions whose validity can be checked against multiple criteria. Our present objective is not to draw biological conclusions. Rather, we want to provide the computational biology community with a tool that allows data of different nature to be integrated in the estimation of the chromatin structure. The software is of public access, see footnote 1, and is sufficiently easy to use, especially by virtue of its graphic interface, which allows the user to load the data files, choose the output format and set most of the parameters relevant to the entire process, from the basic model geometry to the fine algorithmic tuning, with no need to act on the source code.

The ChromStruct strategy to split the chromatin chain into near-isolated blocks to implement a multiscale estimation is also an advantage from the point of view of its computational cost. The choice of making a complete run to generate a single individual of the population of data-compatible configurations and the approximated annealing scheme used to sample the solution space for each block, however, is not guaranteed to provide the most efficient solution to the reconstruction problem. A deeper algorithmic consideration could then lead us to find more efficient solutions without changing the basic strategy. This research direction for the future could also include the introduction of machine learning or deep learning techniques (Caudai et al., 2021a).

## Data Availability

Publicly available datasets were analyzed in this study. This data can be found here: The HI-C data used for those experiments, at a genomic resolution of 5 kbp, refer to human CD34 hematopoietic progenitor cells GM12878 (Dunham et al., 2012), and the data on CTCF-mediated coupling, obtained through ChIA-PET experiments, were downloaded from GEO, accession number GSM1872886 (Tang et al., 2015).

## Footnotes

1 The released versions of ChromStruct are available at https://github.com/ZoeTeti78/ChromStruct/tree/main

2 The HI-C data used for those experiments, at a genomic resolution of 5 kbp, refer to human CD34 hematopoietic progenitor cells GM12878 (Dunham et al., 2012), and the data on CTCF-mediated coupling, obtained through ChIA-PET experiments, were downloaded from GEO, accession number GSM1872886 (Tang et al., 2015).

## References

[B1] Abbas A. He X. Niu J. Zhou B. Zhu G. Ma T. (2019). Integrating Hi-C and FISH data for modeling of the 3D organization of chromosomes. Nat. Commun. 10, 2049. 10.1038/s41467-019-10005-6 31053705 PMC6499832

[B2] Bannister A. J. Kouzarides T. (2011). Regulation of chromatin by histone modifications. Cell. Res. 21, 381–395. 10.1038/cr.2011.22 21321607 PMC3193420

[B3] Caudai C. Galizia A. Geraci F. Le Pera L. Morea V. Salerno E. (2021a). AI applications in functional genomics. Comput. Struct. Biotechnol. J. 19, 5762–5790. 10.1016/j.csbj.2021.10.009 34765093 PMC8566780

[B4] Caudai C. Salerno E. Zoppè M. Merelli I. Tonazzini A. (2019b). ChromStruct 4: a Python code to estimate the chromatin structure from Hi-C data. IEEE/ACM Trans. Comput. Biol. Bioinforma. 16, 1–1878. 10.1109/TCBB.2018.2838669 29993555

[B5] Caudai C. Salerno E. Zoppè M. Tonazzini A. (2015a). Inferring 3D chromatin structure using a multiscale approach based on quaternions. BMC Bioinforma. 16, 234. 10.1186/s12859-015-0667-0 PMC4518643 26220581

[B6] Caudai C. Salerno E. Zoppè M. Tonazzini A. (2019a). Estimation of the spatial chromatin structure based on a multiresolution bead-chain model. IEEE/ACM Trans. Comput. Biol. Bioinforma. 16, 550–559. 10.1109/TCBB.2018.2791439 29994172

[B7] Caudai C. Salerno E. Zoppè M. Tonazzini A. (2015b). “A statistical approach to infer 3D chromatin structure,” in Mathematical models in biology. Editor Zazzu V., (Cham, Switzerland: Springer International Publishing), 161–171.

[B8] Caudai C. Zoppè M. Tonazzini A. Merelli I. Salerno E. (2021b). Integration of multiple resolution data in 3D chromatin reconstruction using ChromStruct. Biology 10, 338. 10.3390/biology10040338 33923796 PMC8072831

[B9] Damaschke N. Gawdzik J. Avilla M. Yang B. Svaren J. Roopra A. (2020). CTCF loss mediates unique DNA hypermethylation landscapes in human cancers. Clin. Epigenetics 12, 80. 10.1186/s13148-020-00869-7 32503656 PMC7275597

[B10] Dixon J. R. Sevaraj S. Yue F. Kim A. Li Y. Shen Y. (2012). Topological domains in mammalian genomes identified by analysis of chromatin interactions. Nature 485, 376–380. 10.1038/nature11082 22495300 PMC3356448

[B11] Duggal G. Patro R. Sefer E. Wang H. Filippova D. Khuller S. (2013). Resolving spatial inconsistencies in chromosome conformation measurements. Algorithms Mol. Biol. AMB 8, 8. 10.1186/1748-7188-8-8 23497444 PMC3655033

[B12] Dunham I. Kundaje A. Aldred S. F. Collins P. J. Davis C. A. Doyle F. (2012). An integrated encyclopedia of DNA elements in the human genome. Nature 489, 57–74. 10.1038/nature11247 22955616 PMC3439153

[B13] Gong H. Ma F. Zhang X. Yang Y. Li M. Chen Z. (2023). A 3D chromosome structure reconstruction method with high resolution Hi-C data using nonlinear dimensionality reduction and divide-and-conquer strategy. IEEE Trans. NanoBioscience 22, 716–727. 10.1109/TNB.2023.3277440 37200118

[B14] Hansen J. C. Connolly M. McDonald C. J. Pan A. Pryamkova A. Ray K. (2018). The 10-nm chromatin fiber and its relationship to interphase chromosome organization. Biochem. Soc. Trans. 46, 67–76. 10.1042/BST20170101 29263138 PMC5818668

[B15] Imakaev M. Fudenberg G. McCord R. P. Naumova N. Goloborodko A. Lajoie B. R. (2012). Iterative correction of Hi-C data reveals hallmarks of chromosome organization. Nat. Methods 9, 999–1003. 10.1038/nMeth.2148 22941365 PMC3816492

[B16] Kapilevich V. Seno S. Matsuda H. Takenaka Y. (2019). Chromatin 3D reconstruction from chromosomal contacts using a genetic algorithm. IEEE/ACM Trans. Comput. Biol. Bioinforma. 16, 1620–1626. 10.1109/TCBB.2018.2814995 29994156

[B17] Li G. Fullwood M. Xu H. Mulawadi F. Velkov S. Vega V. (2009). ChIA-PET tool for comprehensive chromatin interaction analysis with paired-end tag sequencing. Genome Biol. 11, R22. 10.1186/gb-2010-11-2-r22 PMC2872882 20181287

[B18] Lieberman-Aiden E. van Berkum N. L. Williams L. Imakaev M. Ragoczy T. Telling A. (2009). Comprehensive mapping of long-range interactions reveals folding principles of the human genome. Science 326, 289–293. 10.1126/science.1181369 19815776 PMC2858594

[B19] Muhammad I. I. Kong S. L. Akmar Abdullah S. N. Munusamy U. (2020). RNA-seq and ChIP-seq as complementary approaches for comprehension of plant transcriptional regulatory mechanism. Int. J. Mol. Sci. 21, 167. 10.3390/ijms21010167 PMC6981605 31881735

[B20] Nanavaty V. Abrash E. W. Hong C. Park S. Fink E. E. Li Z. (2020). DNA methylation regulates alternative polyadenylation via CTCF and the cohesin complex. Mol. Cell. 78, 752–764.e6. 10.1016/j.molcel.2020.03.024 32333838 PMC7245569

[B21] Oluwadare O. Zhang Y. Cheng J. (2018). A maximum likelihood algorithm for reconstructing 3D structures of human chromosomes from chromosomal contact data. BMC Genomics 19, 161. 10.1186/s12864-018-4546-8 29471801 PMC5824572

[B22] Phillips J. E. Corces V. G. (2009). CTCF: master weaver of the genome. Cell. 137, 1194–1211. 10.1016/j.cell.2009.06.001 19563753 PMC3040116

[B23] Phillips T. (2008). Regulation of transcription and gene expression in eukaryotes. Nat. Educ. 1, 199.

[B24] Tang Z. Luo O. J. Li X. Zheng M. Zhu J. J. Szalaj P. (2015). CTCF-mediated human 3D genome architecture reveals chromatin topology for transcription. Cell. 163, 1611–1627. 10.1016/j.cell.2015.11.024 26686651 PMC4734140

[B25] Trieu T. Oluwadare O. Cheng J. (2019). Hierarchical reconstruction of high-resolution 3D models of large chromosomes. Sci. Rep. 9, 4971. 10.1038/s41598-019-41369-w 30899036 PMC6428844

[B26] Vadnais D. Middleton M. Oluwadare O. (2022). ParticleChromo3D: a particle swarm optimization algorithm for chromosome 3D structure prediction from Hi-C data. BioData Min. 15, 19. 10.1186/s13040-022-00305-x 36131326 PMC9494900

[B27] Wang Z. Gerstein M. Snyder M. (2009). RNA-Seq: a revolutionary tool for transcriptomics. Nat. Rev. Genet. 10, 57–63. 10.1038/nrg2484 19015660 PMC2949280

[B28] Yaffe E. Tanay A. (2011). Probabilistic modeling of Hi-C contact maps eliminates systematic biases to characterize global chromosomal architecture. Nat. Genet. 43, 1059–1065. 10.1038/ng.947 22001755

[B29] Zhu G. Deng W. Hu H. Ma R. Zhang S. Yang J. (2018). Reconstructing spatial organizations of chromosomes through manifold learning. Nucleic Acids Res. 46, e50. 10.1093/nar/gky065 29408992 PMC5934626

